# Lower Lip Reconstruction after Skin Cancer Excision: A Tailored Algorithm for Elderly Patients

**DOI:** 10.3390/jcm13020554

**Published:** 2024-01-18

**Authors:** Raffaele Russo, Paola Pentangelo, Alessandra Ceccaroni, Luigi Losco, Carmine Alfano

**Affiliations:** Plastic Surgery Unit, Department of Medicine, Surgery and Dentistry, University of Salerno, Baronissi, 84081 Salerno, Italy; rafrusso@unisa.it (R.R.); ppentangelo@unisa.it (P.P.); aceccaroni@unisa.it (A.C.); calfano@unisa.it (C.A.)

**Keywords:** lower lip reconstruction, facial reconstruction, single-stage lip reconstruction, local flap, algorithm for lip reconstruction, step technique, squamous cell carcinoma, microstomia

## Abstract

Background: Lower lip reconstruction is crucial to restore oral integrity post-cancer excision. A perfect balance between form and function should be achieved. With an aging demographic, adapting surgical methods to meet the unique needs of the elderly becomes imperative. Our study aims to introduce a specialized algorithm for lower lip reconstruction; it was tailored to geriatric patients and emphasized the use of “simpler flaps”. Additionally, “Pearls and Pitfalls” were provided for surgeons approaching lower lip reconstruction. Methods: Between January 2018 and June 2021, a retrospective study was carried out. Data collection included patient demographics, defect attributes, reconstructive approaches, flap viability assessment, wound healing, and complications. The follow-up was carried out for a period of a minimum of 6 months. Results: Among 78 patients, squamous cell carcinoma predominated with a mean defect area of 3308 cm^2^. Postoperative complications were recorded in two patients. All patients reported sensory restoration and overall satisfaction at the 6-month follow-up; secondary procedures were not necessary. Conclusion: Our reconstructive algorithm, focused on elderly patients, prioritizes less invasive reconstructive techniques and introduces innovative modifications to the established methods to achieve both aesthetic and functional outcomes with a low complication rate. In patients undergoing lower lip reconstruction, the subjective microstomia was found to be less relevant than the objective microstomia.

## 1. Introduction

Reconstructive surgery has witnessed remarkable advancements over the years, offering hope and a renewed quality of life to patients. Among the intricate tapestry of surgical procedures, the reconstruction of the lower lip should achieve a perfect balance between form and function in the human face, aiming to recreate the integrity of the oral region after cancer excision, traumas, or congenital defects [1]. Beyond its aesthetic significance, the lower lip plays a pivotal role in speech, expression, and basic oral functions. Lower lip reconstruction holds a significant place, not only for its functional importance but also for its profound impact on an individual’s self-esteem and social interaction. As the demographics of our population continue to shift, there is a growing need to adapt and refine our surgical approaches to cater to the unique requirements of specific patient groups. Moreover, the novel and widespread scientific trend of a tailor-made and aesthetic “friendly” reconstructive surgery that is far more attentive to donor site morbidity and patients’ discomfort, along with their desires and expectations, should be taken into account [2].

In recent years, the aging population has become a prominent focus within the field of reconstructive surgery [3]. The elderly, often characterized by complex medical histories and age-related changes in tissue quality, present a distinct set of challenges that demand specialized care. Our study aims to fill a notable void in the field by developing a specialized reconstructive “Where is on the lip” algorithm. This was explicitly tailored to elderly patients undergoing lower lip reconstruction and strategically designed to address various lower lip defects based on their size and localization. The effectiveness of less invasive surgical procedures was highlighted, and a specific reconstructive path was detailed for this patient population. Additionally, we aimed to share valuable insights through our “Pearls and Pitfalls” section, offering practical advice and cautionary considerations for surgeons dealing with lower lip reconstruction.

## 2. Patients and Methods

In this retrospective study, patients who underwent lower lip tumor excision and subsequent reconstruction between January 2018 and June 2021 were reviewed. The inclusion criteria encompassed lower lip reconstruction after cancer excision, involvement of vermillion, age > 65 years, a minimum follow-up of 6 months, the ability to understand and sign informed consent, and the ability to attend the follow-up visits. Exclusion criteria included subtotal or total defects requiring microsurgical techniques, multiple-stage procedures, a lack of documentation, and a loss to follow-up (Table 1).

The study was conducted in accordance with the ethical principles outlined in the Declaration of Helsinki and its later amendment, and all participating patients provided written informed consent, which included a section granting permission for the use of photographs. Demographic data were collected; a histological diagnosis and a macroscopical evaluation of the defect (size, morphology, localization, color, and adjacent structures involved) were performed; surgical techniques were employed, and complications and cancer recurrence were also recorded. In cases where lymph node involvement was suspected upon clinical evaluation, tumors exceeded a size of 3 cm, or lesions displayed signs of potential invasiveness, an ultrasound examination of the draining lymph nodes was carried out. Subsequently, additional diagnostic investigations, such as computed tomography (CT) scans or other advanced diagnostic tests were conducted as per the recommendations of the multidisciplinary oncology team.

During the first postoperative visit, follow-up pictures were taken, and the patient was examined. Differences in skin pigmentation among the reconstructed area and adjacent regions and altered contours of the reconstructed or donor site were evaluated. The lack of functionality in the perioral region or drooling and asymmetries were recorded during later visits. All patients were evaluated after 1 and 3 days post-surgery to assess flap viability and wound healing as well as the presence of complications like bleeding, hematoma, or wound dehiscence <1 cm and >1 cm. Then, postoperative follow-up visits were planned after 7, 15, and 30 days. After 1 month, and in the late follow-up period, any alteration of lip functionality was assessed by considering 3 main aspects: static lip functionality, dynamic lip functionality (during food and drink intake), and alteration in phonation. For the assessment of potential microstomia, the valuable scale proposed by Shaikh et al. [4] was used. This scale comprises both subjective parameters reported by the patients and objective parameters recorded by the medical staff during follow-up visits (Table 2).

At a minimum follow-up duration of 6 months, postoperative photographs were taken, and patients underwent comprehensive re-evaluation, encompassing photographic analysis, an assessment of the aesthetic outcomes, and an evaluation of static and dynamic lip functionality (Table 3).

## 3. Surgical Technique

Before surgery, preoperatory IV prophylaxis with cefazoline (2 g) was administered to all patients. Whenever possible, the procedures were carried out under local anesthesia and epinephrine. Larger surgeries were performed under general anesthesia, although preoperatory skin infiltration was still performed to detach the upper from the lower anatomical layers and perform an easier dissection. In all cases treated under general anesthesia, patients underwent a perioperative hydration therapy within 24 h, consisting of 500 cc of electrolyte rehydration solution and 1500 cc of 0.9% NaCl saline solution. In cases of local anesthesia, patients received therapy with 1000 cc of 0.9% NaCl saline solution and 500 cc of electrolyte rehydration solution. 

**Figure 1:**
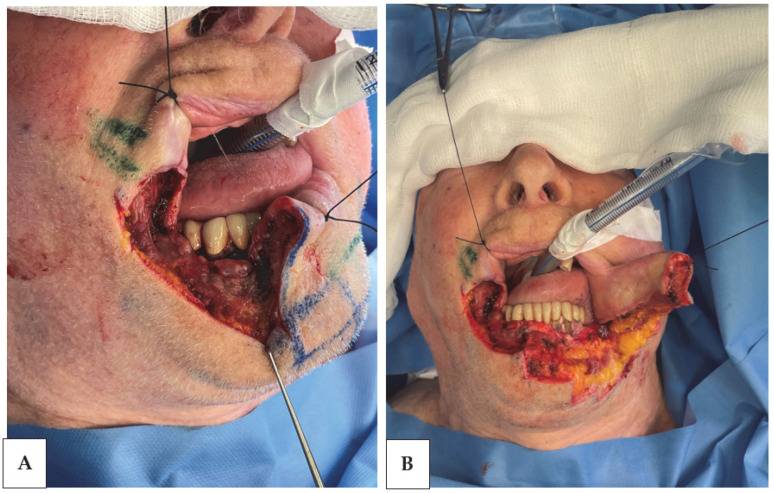
Eighty-nine-year-old male patient who underwent cancer excision followed by reconstruction using the asymmetric step technique: (**A**) Primary defect. Note the presence of the full-thickness silk suture positioned at the level of the patient’s vermilion. (**B**) Raised flap. The silk suture is an aid for traction and a guide for subsequent approximation.

The established excision parameters for primary lesions were set at a minimum of ≥0.6 cm. After anesthesia was administered, to control bleeding and enhance surgical precision, a 0-silk ligature was placed along the lip on both sides 1 cm beyond the oncologic resection margin. This ligature was strategically passed just below the red vermilion border and through the mucosal tissue (traction ligature) (Figure 1). Regarding the preoperative planning and surgical techniques used, several factors were taken into account, including the lesion’s location on the lip, the potential involvement of the commissure, and the extent of the lesion. We also adhered to the principle of the “Reconstruction Ladder”, which entails beginning with the simplest techniques and progressing to more complex ones as needed [5].

In any case, before suturing, a “test” suture was placed at the junction between the vermillion border and the white lip without tying it to bring the lip margins into close approximation and simulate the final closure. After achieving optimal lip positioning, a watertight closure was performed, and particular attention was paid to the separate reconstruction of the various anatomical layers of the lip. First, the mucosa was sutured using absorbable Vicryl rapide 4 or 5-0 stitches. Subsequently, Vicryl 4-0 was employed to restore the integrity of the orbicular muscle and subcutaneous tissue. Finally, the skin was sutured using 5- or 6-0 polypropylene. Sutures were typically removed approximately 10–12 days post-operation. However, it is essential to note that the specific timing of suture removal was led by the clinical findings obtained during follow-up visits.

### 3.1. Pearls and Pitfalls

- Prior to excision, a meticulous technique involving the placement of a 0-silk ligature is employed; it was previously introduced as a “traction ligature” (Figure 1).

This suture spans the entire thickness of the lip, extending from the mucosa to the skin–vermillion border. The primary objective is to streamline the identification and meticulous alignment of the vermilion’s red margins, ensuring symmetrical outcomes during the reconstructive phase. The utilization of this suture presents two pivotal advantages. Firstly, it plays a crucial role in achieving flawless alignment. Secondly, this “full thickness” suture also serves the functional purpose of minimizing bleeding during the dissection phase, providing enhanced traction for a more accurate incision and superior control over tissue margins during subsequent reconstructive procedures.

- During the excision process, our approach diverges from some authors who choose to preserve the mucosal layer, especially in less extensive defects. We opt for a more aggressive stance, creating a full-thickness defect every time the vermillion is involved. This approach ensures not only a higher degree of oncological radicality [6] but also a pleasing labial contour without redundancies.
- On the superficial layer, a vertical mattress suture [7,8] is often preferred for the suture of vermillion to achieve margin eversion and prevent invagination. The cutaneous sutures are applied as interrupted simple stitches, employing the vertical mattress technique “on demand” to prevent invagination in areas that may appear to be prone. Furthermore, Allgöwer–Donati [7] stitches are the chosen technique to suture the corners of the flaps. Within the mucosa, Vicryl rapide 4-0 is employed to reduce patient discomfort.

### 3.2. Statistical Analysis

A statistical analysis was conducted using IBM SPSS Statistics for Macintosh, Version 28.0 software (IBM Corp., Armonk, New York, NY, USA). The values for categorical variables were analyzed using a chi-squared test; the values for quantitative variables were analyzed using a two-tailed Mann–Whitney test. Significance was set at a value of *p* < 0.05.

## 4. Results

In the period spanning from January 2018 to June 2021, a cohort comprising 85 patients underwent lower lip reconstruction due to tumor excision. Seven patients within this cohort were excluded from our study due to exitus unrelated to lip cancer or surgical procedures, multiple-step reconstruction, or loss to follow-up. Among the 78 patients who were deemed eligible for inclusion in our comprehensive analysis, 62.8% were male and 37.2% female. The collective mean age of these patients was 81.5 years, with ages ranging from a minimum of 65 years to a maximum of 94 years.

Regarding additional patient demographics, it is noteworthy that 32 patients were identified as active smokers, constituting 41% of our study cohort. None of the subjects presented with concomitant diabetes. However, it is of significance to highlight that a subset of 42 patients, accounting for approximately 53.8% of the study population, had cardiovascular disease and were, consequently, undergoing anticoagulant/antiaggregant and/or antihypertensive therapy.

Within the context of etiological considerations for lower lip reconstruction, squamous cell carcinoma was the predominant causative factor, contributing to 64 (82%) of the total reconstructed lips. Subsequently, basal cell carcinoma emerged as the second most prevalent etiological entity, manifesting in 11 cases (14.1%). Other lesions accounted for three cases (3.8%) of the reconstructed lips cases under analysis (two cases of lichenoid keratosis and one case of angioma) (Table 4).

After tumor resection, 31 defects exhibited the greatest dimension of 2 cm or less; meanwhile, 47 measured between 2 and 4 cm in the greatest dimension (Table 5). T classification is provided in Table 6.

Among the diverse approaches, wedge resection emerged as the most frequently employed technique (30.7% of cases), characterized by a relatively brief average operation time of 28 min and a mean defect size of 2.38 cm^2^. It is noteworthy that the majority of these wedge excisions (21 out of 24) were V-shaped, with the remaining three being W-shaped. The step technique flap showcased an extended average operation time of 45.2 min with a mean defect size of 3.2 cm^2^. Noteworthy findings included the Karapandzic flap, which required a substantial average operation time of 85.4 min and yielded a mean defect size of 3.9 cm^2^. Additionally, the Gillies fan flap and Estlander flap, either alone or in conjunction with a step technique, demonstrated unique aspects with varying operation times and mean defect sizes. The Estlander flap stood out with a concise average operation time of 44.7 min and a mean defect size of 3.7 cm^2^. Meanwhile, the Webster flap was associated with a notably prolonged average operation time of 88.2 min and a larger mean defect size of 4.4 cm^2^ (Table 7).

Out of the total patient population, three individuals exhibited lymph node involvement; moreover, among them, two underwent lymph node biopsy or laterocervical lymph node dissection. One patient was deemed unfit for lymphadenectomy and underwent subsequent chemotherapy.

No major complications (i.e., pulmonary embolism, need for blood transfusion, etc.) were recorded. All harvested flaps were vital upon postoperative evaluation. In our series, we did not report any perioperative bleeding events, total flap necrosis, hematomas, or infections. Notably, fluid administration and operation duration did not show a significant correlation with the complications observed in our study (*p* > 0.05). 

In two cases, specifically, one involving the step technique and another utilizing the Karapandzic flap, a minor margin dehiscence of less than 1 cm was observed at 12 and 15 days postoperatively, respectively. These cases did not necessitate reintervention and healed through secondary intention within 1 month following the procedure. None of the patients within our cohort required any form of surgical revision. The presence of objective microstomia was observed at the 6-month follow-up in two patients who underwent wedge excision, three patients treated with the step technique, thirteen patients who underwent Karapandzic flap reconstruction, five patients with Gillies flap reconstruction, four patients who underwent a combination of the Estlander flap + step technique, one patient who had Estlander flap reconstruction, and four patients who underwent Bernard Burow modified by Webster flap reconstruction. In all cases, patients reported sensory recovery at the 6-month follow-up (Table 8 and Table 9). The association between complication rates and patients’ demographics, including smoking status and use of anticoagulant medications was examined, yielding results that were not statistically significant (all *p* > 0.05).

## 5. Discussion

Head and neck squamous cell carcinoma is a major pathological type among head and neck cancers [9,10]. Lip cancer is a common type of oral tumor that more commonly affects Caucasian men who smoke and are between the ages of 50 and 70 [11]. Squamous cell carcinoma accounts for more than 90% of lip cancers; it occurs more often on the lower lip, followed by basal cell carcinoma, which occurs more often on the upper lip, and, unlike the previous one, it is usually not associated with lymph node metastasis [12,13].

Although the scientific literature depicted various surgical procedures and approaches for facial unit reconstruction after cancer excision [14,15,16,17], there has been limited research focusing on the intricacies of lip reconstruction in elderly patients. This subset of patients presents unique challenges and characteristics that, in our opinion, make the reconstructive process different from that of the general population. 

In clinical practice, it is imperative to avoid the misconception that skin laxity in elderly patients is equivalent to that in younger counterparts [3]. Elderly patients, owing to their increased skin laxity, afford greater flexibility in the use of technically simpler flaps, leading to a reduction in surgical time, quicker functional recovery, and a reduction in surgical risks, as supported by the scientific literature [18]. This patient population often exhibits less efficient wound healing compared to their younger counterparts, resulting in the general tendency of having less exuberant scarring and better overall scar outcomes, offering a superior aesthetic result with equivalent surgical techniques [19,20]. On the other hand, it is essential to emphasize that elderly patients frequently exhibit significant comorbidities which make it imperative to perform procedures under local anesthesia. Moreover, particularly in the presence of diabetic patients, the healing time could be prolonged [21,22], requiring heightened vigilance during post-operatory surgical wound care to preempt the risk of complications and detect any potential issues at an earlier stage.

**Figure 2:**
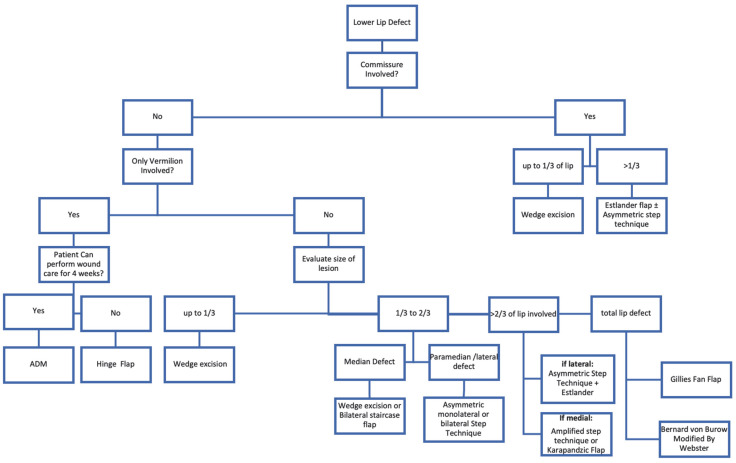
Algorithm for lip reconstruction: A structured approach that considers the location and size of the defect. ADM: Acellular Dermal Matrix.

Taking these premises into consideration, we have developed a reconstructive algorithm for the reconstruction of lower lip defects following tumor excision in elderly patients: this allowed us to prioritize reconstruction using less invasive surgical techniques (Figure 2). This approach reduces surgical time and the risk of complications associated with surgical stress in elderly patients while achieving outcomes that are more than acceptable from both an aesthetic and functional standpoint.

Seventy-eight lower lip reconstructions were performed at our institution for defects, ranging from less than 1/3 of the lip to more than 2/3 of the lip. Following our reconstructive flowchart, defects involving up to 1/3 of the lower lip, regardless of their location, were approached with a wedge resection (24 cases); this approach was also extended to defects affecting as much as 40% of the lower lip length, provided they did not involve the commissure. This procedure is consistent with the findings of Soliman et al. [23]. As described in their study on post-Mohs surgery reconstruction, they suggest that superior aesthetic outcomes can be achieved with defects exceeding 50 percent of the lower lip compared to more aggressive techniques.

**Figure 3:**
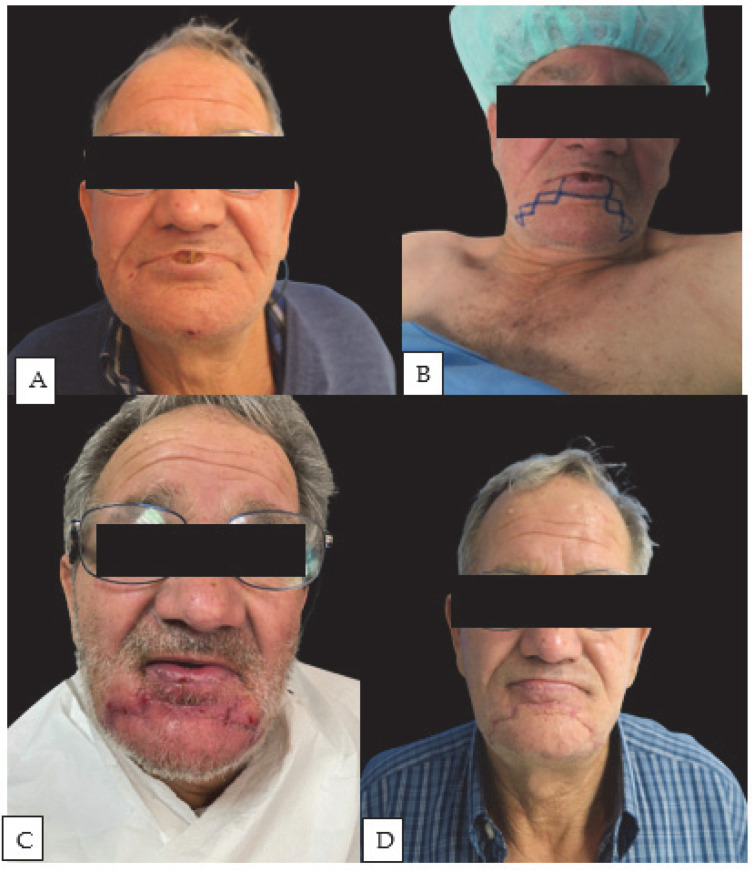
Bilateral symmetric step technique reconstruction on a 68-year-old patient: (**A**) preoperative picture; (**B**) preoperative drawing of symmetric modified step technique; (**C**) 10 days post-op; (**D**) six months post-op follow-up—note the absence of microstomia even though the defect involved >2/3 of the lip.

The second most frequently employed flap, which has shown an excellent balance between aesthetic functionality and ease of execution, is the step technique. In the original description by Johanson et al. (1974), the step incisions traversed the full thickness of the lower lip [24]. The rectangles below the steps were also excised through the complete thickness of the lip [25]. In our series, the step technique is different from the original description: the surgical indication was extended beyond the usual boundaries placed for younger patients [26]. Specifically, a broader and more extensive design was planned, especially for defects that encompass 2/3 of the lip length. The design transcends the typically recommended anatomical boundary of the nasolabial groove to facilitate greater tissue recruitment, which is essential for lower lip reconstruction, thus reducing the risk of microstomia (Figure 3).

Unlike the original technique, where peripheral portions underwent full-thickness excision, the resection was limited to the skin only in the outermost margins of the modified flap to avoid any damage to the mental nerve [10,27,28].

Moreover, to broaden the scope of eligible patients with substantial paramedian and lateral defects, irrespective of commissure involvement, the step technique was associated with the Estlander flap. We favored this approach for cases in which the use of the step technique alone could not yield the desired aesthetic or functional outcomes or the development of microstomia. For subtotal defects of the lip, especially in elderly patients with limited skin laxity that did not permit us to consider less invasive reconstructive options, we turned to more complex and extensive flaps such as the Karapandzic flap, which was employed for the reconstruction of subtotal defects in 13 patients, while the remaining subset of patients with subtotal lip defects were treated with the Gillies flap, in addition to the Webster flap, for a total of 12 patients: six were treated with Webster flap and six with the Gillies flap. 

The substantial variation in lesion sizes observed in our study, with an average of 3.308 cm^2^, and a significant subset of patients presenting with lesions exceeding 2 cm (60.2% of our cohort), deviates from the expected distribution reported in the existing literature [26]. This apparent discrepancy could be attributed to several noteworthy factors. Firstly, the onset of our study during the peak of the COVID-19 pandemic introduced an unprecedented and complex set of circumstances. As the literature has extensively documented, the fear of contracting the virus within healthcare facilities contributed to a critical delay in the diagnosis and treatment of various medical conditions, including lip lesions [29,30]. Patients were hesitant to seek medical attention, and this reluctance to engage with healthcare institutions led to a significant postponement in the diagnosis and management of their conditions. Consequently, patients who might have sought timely medical advice for smaller lesions allowed their conditions to progress, resulting in larger and more advanced lesions by the time they eventually presented for treatment [31,32,33]. Secondly, a noteworthy aspect of our patient population is their predominantly elderly demographic. This age group is known to exhibit a higher propensity to neglect their health concerns and endure symptoms without seeking medical consultation [32].

The most prevalent histological type was squamous cell carcinoma (64 patients, 82%). This is in line with the current literature [11]; the second most common cause was basal cell carcinoma, 11 cases (14.1%).

Two cases of incomplete excision were reported and were included as separate cases. More specifically, the wedge excision technique was initially employed in both cases: these patients underwent reoperation, once again undergoing wedge excision, obtaining histologically disease-free margins. In two instances, specifically, one in which the Karapandzic technique was used and another that utilized the step technique, minor margin dehiscence <1 cm was observed. It is noteworthy that these cases did not necessitate surgical intervention and, instead, exhibited a successful resolution through secondary intention healing. None of the patients within our cohort required any form of surgical revision.

In our study, two cases of dehiscence post-surgery were recorded with no instances of bleeding or infection. Gudzhabidze et al. [34] reported local complications in 23.1% of cases after surgical treatment for lower lip cancer. The most frequent complication noted was secondary wound repair along with the formation of an oral fistula. In their series, flap necrosis was detected in 8.2% of cases, predominantly in patients where the flap was harvested from the upper lip. 

Our study results suggest a lower incidence of local complications, specifically dehiscence, compared to the findings reported by Gudzhabidze et al. The absence of bleeding and infection in our cases highlights potential variations in complication profiles between different surgical approaches or patient populations. Anyhow, further comparative analysis is warranted to discern contributing factors to these observed differences.

In our study, the presence of objective microstomia at the 6-month follow-up was observed in a subset of patients who underwent various reconstructive techniques. Notably, two patients who underwent wedge excision, three patients treated with the step technique, thirteen patients undergoing Karapandzic flap reconstruction, five patients with Gillies flap reconstruction, four patients who underwent a combination of the Estlander flap + step technique, one patient undergoing Estlander flap reconstruction, and four patients who underwent Bernard Burow modified by Webster flap reconstruction exhibited objective microstomia. This observation may be viewed as a potential limitation of our approach, which, at times, extends beyond the classical indications for excision. However, what is particularly intriguing is the subjective perception of this condition among the patients. When asked about the presence of subjective microstomia, their responses revealed a significant discrepancy. Only a minority of patients who objectively exhibited microstomia acknowledged its presence when questioned directly. Specifically, one patient who underwent wedge excision, one patient who was treated with the step technique, nine patients who underwent Karapandzic flap reconstruction, three patients with Gillies flap reconstruction, one patient with Estlander flap reconstruction, and four patients who underwent Bernard Burow modified by Webster flap reconstruction were subjectively affected by microstomia according to our criteria (Table 7). 

This incongruity between objective measurements and subjective patient perception raises crucial questions about the impact of microstomia on functional outcomes and patients’ quality of life. It is evident that, while some patients objectively exhibit a reduced oral aperture, they subjectively perceive their overall quality of life as satisfactory because they have successfully adapted to the physical limitations posed by microstomia and are satisfied with their functional abilities. These same patients, when offered the option of commissuroplasty revision, declined the procedure. Their rationale for refusal was rooted in their perceived high quality of life and overall aesthetic satisfaction. These findings underscore the importance of considering not only objective clinical outcomes but also the patient’s perception and adaptation to their post-reconstruction condition. It emphasizes the need for individualized patient-centered care, considering not only the physical results of the procedure but also the patient’s own assessment of their well-being. Further research in this area is warranted to gain a deeper understanding of the psychological and functional aspects of microstomia in lip reconstruction patients and to optimize patient care and satisfaction.

Shaikh et al. [4] conducted a lip reconstruction study utilizing the same scale employed in our research. In their cohort of 21 reconstructions, 14.3% demonstrated subjective microstomia, and 57.2% exhibited objective microstomia. In our larger cohort, 19.3% subjective microstomia and 24.9% objective microstomia were observed. The increased prevalence of subjective microstomia in our study may be attributed to both the larger sample size and our deliberate extension beyond conventional lower lip reconstruction approaches.

Moreover, it is noteworthy that Shaikh et al. included microsurgical reconstructions, a subset we intentionally excluded. This exclusion may contribute to the observed differences in microstomia rates compared to our study. 

In two cases, despite obtaining cancer-free margins on histological examination, subsequent follow-up visits revealed disease recurrence. In the first case, a patient with basal cell carcinoma who had undergone wedge excision exhibited recurrence contiguous to the excision margin and was managed with a new wedge excision. In the second case, a patient with squamous cell carcinoma treated with an asymmetric step technique exhibited a recurrence in the chin region, which was addressed through surgical excision and flap reconstruction with a bilateral advancement flap.

In our surgical algorithm, as indicated by the exclusion criteria, we deliberately excluded flaps in a two-step procedure like the Abbe flap to avoid subjecting the patient to a two-step surgery. Sanniec et al., in their interesting article on post-Mohs surgery lip reconstruction, did not find significant utility in using the Abbe flap for lower lip defect reconstruction [6]. We align with these authors as we also prefer an approach that minimizes the interruption of the continuity of the upper lip for the reconstruction of lower lip defects whenever possible. The rationale behind our choice is rooted in the fact that, especially in more complex reconstructions requiring large flaps, there is inevitably a disruption of the anatomy of the lower lip. Therefore, we aim to preserve the physiological sphincter function, at least in the upper lip, whenever possible.

Our study has notable limitations, including the exclusion of free flaps, which, although complex and carry a high risk in elderly patients with comorbidities, can be an excellent reconstructive option when performed by an experienced microsurgeon. These procedures are particularly effective for patients with defects exceeding 80% of the lower lip with involvement of the chin region [4,35,36,37].

The small sample size is another non-negligible limitation, and this could be the reason for the lack of significant results. Additionally, the lack of inclusion and classification of microstomia severity in patients with prostheses highlights the need for further investigation and clarification in future research. These limitations emphasize the importance of future prospective research with larger and more diverse cohorts to provide robust evidence for informed clinical decision-making in lip reconstruction.

## 6. Conclusions

Our reconstructive algorithm, focused on elderly patients, prioritizes less invasive reconstructive techniques and introduces innovative modifications to the established methods: it is a summary of the delicate balance between aesthetic/functional outcomes and surgical feasibility with low complication rates. The subjective microstomia was found to be less relevant than objective microstomia in our patients’ cohort, emphasizing the importance of individualized, patient-centered care.

## Figures and Tables

**Table 1:** Inclusion and exclusion criteria.

Inclusion Criteria	Exclusion Criteria
Reconstruction after cancer excision	Microsurgical technique
Involvement of vermillion	Multiple stages reconstruction
Age > 65 years	Lack of documentation
Follow-up > 6 months	Loss to follow-up

**Table 2:** Criteria for microstomia assessment.

	Criteria	
	Subjective	Objective
Normal	No problems eating. Can put tablespoon in mouth. Satisfactory oral hygiene.	Both commissure equidistant to NLF and at the level of a vertical line departing from the medial cantus (medial limbus)
Moderate	No problems eating. Can put teaspoon in mouth. Satisfactory oral hygiene.	One or both commissure lateral to alar groove but medial to medial limbus
Severe	Problems eating. Cannot put teaspoon in mouth. Problems with oral hygiene.	One or both commissure medial to alar groove

Note. Adapted from Shaikh AI et al. [4]. The functional and aesthetic outcome of different methods of reconstruction of full-thickness lip GMS Interdiscip Plast Reconstr Surg DGPW. 2022.

**Table 3:** Study design.

	V1	1 d	3 d	7 d	15 d	30 d	3 m	6 m
Informed consent	●							
Evaluation of admission criteria	●	●	●	●	●	●	●	●
Demographic and anamnestic data	●							
Photographs	●	●	●	●	●	●	●	●
Associated therapies	●	●	●	●	●	●	●	●
Vitality of the flap		●	●	●				
Wound healing		●	●	●	●	●		
Complications		●	●	●	●	●	●	●
Lip functionality *					●	●	●	●

Note: * Lip functionality intended in both static and dynamic movement. V1. First visit; 1 d: one day post-surgery; 3 d: three days post-surgery; 7 d: seven days post-surgery; 15 d: fifteen days post-surgery; 30 d: thirty days post-surgery; 3 m: three months post-surgery; 6 m: six months post-surgery.

**Table 4:** Patients’ demographics.

Variable	*n* (%)
Patients	78
Mean age, years	81.5
Gender – Male – Female	49 (62.8%) 29 (37.2%)
Smoking habit – Smokers – Non-smokers	32 (41.0%) 46 (58.9%)
Anticoagulant assumption – Yes – No	42 (53.8%) 36 (45.1%)
Histology – Squamous cell carcinoma – Basal cell carcinoma – Other lesions	64 (82%) 11 (14.1%) 3 (3.8%)

**Table 5:** Size and location of defect.

Variable	*n* (%)
Mean area of defect, cm^2^	3308 cm^2^
Area of defect, cm^2^ – <1 cm^2^ – 1 > 2 cm^2^ – >2 cm^2^	10 (12.8%) 21 (26.9%) 47 (50.1%)

**Table 6:** T classification.

Variable	*n* (%)
Area of lesion, cm^2^ – T1 – T2 – T3	16 (20.5%) 27 (34.6%) 34 (43.6%)
– T4	1 (1.2%)

**Table 7:** Total number of patients per surgical procedure and average operation time.

Technique	Total Patients, *n* (%)	Average Operation Time (±SD)	Mean Defect Size, cm^2^ (±SD)
Wedge Resection	24 (30.7%)	28 (±2.7 min)	2.38 (±0.36)
Step Technique Flap	16 (20.5%)	45.2 (±4.4 min)	3.2 (±0.2)
Karapandzic Flap	13 (16.6%)	85.4 (±3.8 min)	3.9 (±0.2)
Gillies Fan Flap	6 (7.6%)	84 (±4.4 min)	3.6 (±0.2)
Estlander Flap + Step	7 (8.9%)	69.4 (±10.3 min)	4.1 (±0.1)
Estlander Flap	6 (7.6%)	44.7 (±1.4 min)	3.7 (±0.1)
Webster flap	6 (7.6%)	88.2 (±1.5 min)	4.4 (±0.1)

**Table 8:** Summary of complications.

Complication	*n* (%)
Infection	0 (0%)
Recurrence	2 (1.5%)
Subjective Microstomia	22 (19.3%)
Objective Microstomia	32 (24.9%)
Wound dehiscence	2 (1.5%)
Flap failure	0 (0%)
Bleeding	0 (0%)
Hypoesthesia *	0 (0%)

* Hypoesthesia 6 months after surgery.

**Table 9:** Microstomia assessment based on different techniques.

Technique	Subjective Microstomia, *n* (%)	Objective Microstomia, *n* (%)
Wedge Resection	1 (4.2%)	2 (8.3%)
Step Technique Flap	1 (6.3%)	3 (16.8%)
Karapandzic Flap	9 (69.2%)	13 (100%)
Gillies Fan Flap	3 (50%%)	5 (83.3%)
Estlander Flap + Step Technique	3 (42.8%)	4 (57.1%)
Estlander Flap	1 (16.6%)	1 (16.6%)
Webster flap	4 (66.6%)	4 (66.6%)

## Data Availability

The data presented in this study are available upon request from the corresponding author. The data are not publicly available due to privacy restrictions.
